# Fungal signature differentiates alcohol-associated liver disease from nonalcoholic fatty liver disease

**DOI:** 10.1080/19490976.2024.2307586

**Published:** 2024-02-01

**Authors:** Greta Viebahn, Phillipp Hartmann, Sonja Lang, Münevver Demir, Xinlian Zhang, Derrick E. Fouts, Peter Stärkel, Bernd Schnabl

**Affiliations:** a Department of Medicine, University of California San Diego, La Jolla, CA, USA; b Department of Pediatrics, University of California San Diego, La Jolla, CA, USA; c Division of Gastroenterology, Hepatology & Nutrition, Rady Children’s Hospital San Diego, San Diego, CA, USA; d Faculty of Medicine, University of Cologne, Cologne, Germany; e Department of Hepatology and Gastroenterology, Campus Virchow Clinic and Campus Charité Mitte, Charité University Medicine, Berlin, Germany; f Division of Biostatistics and Bioinformatics, Herbert Wertheim School of Public Health and Human Longevity Science, University of California San Diego, La Jolla, CA, USA; g Department of Genomic Medicine, J. Craig Venter Institute, Rockville, MD, USA; h Université Catholique de Louvain, St. Luc University Hospital, Brussels, Belgium; i Department of Medicine, VA San Diego Healthcare System, San Diego, CA, USA

**Keywords:** Mycobiome, microbiome, fungi, ALD, NAFLD

## Abstract

The fungal microbiota plays an important role in the pathogenesis of alcohol-associated liver disease (ALD) and nonalcoholic fatty liver disease (NAFLD). In this study, we aimed to compare changes of the fecal fungal microbiota between patients with ALD and NAFLD and to elucidate patterns in different disease stages between the two conditions. We analyzed fungal internal transcribed spacer 2 (ITS2) sequencing using fecal samples from a cohort of 48 patients with ALD, 78 patients with NAFLD, and 34 controls. The fungal microbiota differed significantly between ALD and NAFLD. The genera *Saccharomyces*, *Kluyveromyces*, *Scopulariopsis*, and the species *Candida albicans* (*C. albicans*), *Malassezia restricta* (*M. restricta*), *Scopulariopsis cordiae* (*S. cordiae*) were significantly increased in patients with ALD, whereas the genera *Kazachstania* and *Mucor* were significantly increased in the NAFLD cohort. We identified the fungal signature consisting of *Scopulariopsis*, *Kluyveromyces*, *M. restricta*, and *Mucor* to have the highest discriminative ability to detect ALD vs NAFLD with an area under the curve (AUC) of 0.93. When stratifying the ALD and NAFLD cohorts by fibrosis severity, the fungal signature with the highest AUC of 0.92 to distinguish ALD F0-F1 vs NAFLD F0-F1 comprised *Scopulariopsis*, *Kluyveromyces*, *Mucor*, *M. restricta*, and *Kazachstania*. For more advanced fibrosis stages (F2-F4), the fungal signature composed of *Scopulariopsis*, *Kluyveromyces*, *Mucor*, and *M. restricta* achieved the highest AUC of 0.99 to differentiate ALD from NAFLD. This is the first study to identify a fungal signature to differentiate two metabolic fatty liver diseases from each other, specifically ALD from NAFLD. This might have clinical utility in unclear cases and might hence help shape treatment approaches. However, larger studies are required to validate this fungal signature in other populations of ALD and NAFLD.

## Introduction

Alcohol-associated liver disease (ALD) and nonalcoholic fatty liver disease (NAFLD) are associated with significant morbidity and mortality and continue to become more common worldwide.^1–4^ The prevalence of ALD is estimated to be 4.8% globally and that of NAFLD 30% globally.^1,4^ Better noninvasive biomarkers are required to more easily detect early and more advanced stages of liver disease.^5,6^ One noninvasive biomarker is the intestinal microbiota. The gut microbiota plays a role in many liver diseases, as evidenced by studies with germfree and conventional mice.^7^ Specific bacterial signatures for the diagnosis of advanced fibrosis and cirrhosis were recently identified for NAFLD.^8,9^

We previously investigated the role of the fungal microbiome, or mycobiome, in ALD^10–12^ and NAFLD.^13^ Since a number of similar shifts of the fungal microbiome are associated with higher severity of both ALD and NAFLD, such as increased fecal proportions of *Candida albicans* (*C. albicans*),^10–13^ we now aimed to compare the changes of the fungal microbiota between ALD and NAFLD and to elucidate patterns in different disease stages between the two conditions.

## Material and methods

### Patients

Our patient cohort and study design has been previously described in detail.^12–14^ In brief, our cohort consisted of 34 control patients, 58 alcohol-associated liver disease (ALD) patients, and 78 nonalcoholic fatty liver disease (NAFLD) patients. All ALD patients were heavy drinkers consuming over 60 g of alcohol per day for more than 1 year and had associated liver disease as evidenced by serum aspartate aminotransferase (AST) level >40, alanine aminotransferase (ALT) level >40, controlled attenuation parameter (CAP) per FibroScan >250 dB/m (presence of steatosis further confirmed by Doppler ultrasound), liver stiffness measurement per FibroScan ≥7.6 kPa,^15^ and/or serum caspase-cleaved and intact cytokeratin 18 (CK18-M65) ≥266.^16^ Patients with alcohol use disorder (AUD) without evidence of ALD have been excluded for our analysis. The patients with ALD were prospectively enrolled at St. Luc University Hospital in Brussels, Belgium from April 2017 to January 2019, where they were admitted for a highly standardized and controlled 3-week detoxification and rehabilitation program, during which a FibroScan was performed, and a fasting blood sample was collected on the day of admission. Stool samples were obtained from the first bowl movement after admission. Exclusion criteria included use of antibiotics, probiotics, or prebiotics during the 2 months preceding enrollment, use of immunosuppressive medications, diabetes, inflammatory bowel disease, known liver disease of any other etiology, or clinically significant cardio-vascular, pulmonary, or renal co-morbidities, and age <18 y. NAFLD patients, diagnosed by the presence of steatosis in >5% of hepatocytes on liver biopsy or by clinical, laboratory, and imaging findings consistent with cirrhosis, were prospectively enrolled at the University Hospital of Cologne in Cologne, Germany from March 2015 to December 2018. Exclusion criteria included antibiotic use within 6 months prior to the study, known malignancy, pregnancy, and age <18 y. These subjects were compared with healthy volunteers enrolled in Cologne, Germany (*n* = 16) and Brussels, Belgium (*n* = 18), matched for gender, age, and body mass index (BMI), who drank less than 20 g of alcohol per day. Clinical, demographic and microbiota-related data from ALD patients and NAFLD patients have been reported upon in prior studies.^12–14^

### Ethics

The study protocol conforms to the ethical guidelines of the 1975 Declaration of Helsinki and was approved by the institution’s human research and ethical committee (Université Catholique de Louvain, Brussels, Belgium; B403201422657 and the local Ethics Committee at the University of Cologne, Germany; # 15–056), as previously described, and patients were enrolled after written informed consent was obtained.^12–14^

### Serum biomarkers

All blood samples were collected under fasting conditions. ALD patient blood samples were tested at the clinical laboratory associated with St. Luc University Hospital, Brussels, Belgium. NAFLD patient blood samples were tested at the University Hospital of Cologne, Germany.^12–14^

### Liver stiffness and steatosis measurement

Vibration-controlled transient elastography (FibroScan, Echosens, Paris, France) was performed in fasting patients by experienced operators, blinded to all clinical patient data. At least 10 valid measurements were performed, and the median value of these measurements was reported in kPa. Patients were first scanned with the M probe, and if indicated by the equipment, patients were rescanned with the XL probe, in accordance with the manufacturer’s protocol. Liver stiffness measurement cutoff of 7.6 kPa was used to discriminate mild fibrosis (stage F0-F1) from significant fibrosis (stage F2-F4) and controlled attenuation parameter cutoff of 250 dB/m was used for significant steatosis.^15,17,18^

### Fecal DNA extraction, fungal sequencing, and bioinformatic processing of ITS2 sequences

Internal transcribed spacer 2 (ITS2) sequencing data have been reported before^12,13^ and have been re-analyzed for this analysis. In brief, fecal DNA was extracted using the DNA fast stool mini kit (Qiagen, Hilden, Germany) according to the manufacturer’s protocol, as described.^12,13,19^ Before DNA extraction, bead beating of fecal samples with lysis buffer was performed using 0.7 mm garnet PowerBead tubes (Qiagen, Hilden, Germany). Bead beating was performed using the BioSpec Mini-BeadBeater 96 for 2 × 30 seconds at 50 Hz. PCR and sequencing of the ITS2 genomic region was performed as previously described using the following primer pair (*italics* = overhang adapter sequence, **bold** = region-specific sequence): 5.8S-Fun (read 1) [*TCGTCGGCAGCGTCAGATGTGTATAAGAGACAG***AACTTTYRRCAAYGGATCWCT**] and ITS4-Fun (read 2) [*GTCTCGTGGGCTCGGAGATGTGTATAAGAGACAG***GCCTCCGCTTATTGATATGCTTAART**],^20^ using Illumina’s Fungal Metagenomic Sequencing Demonstrated Protocol (https://support.illumina.com/downloads/fungal-metagenomic-sequencing-demonstrated-protocol-1000000064940.html). Amplification was performed using KAPA HiFi HotStart ReadyMix (Thermo Fisher Scientific, Waltham, USA). Illumina indices and sequencing adaptors were attached using the Nextera® XT v2 Index Kit following the Illumina ITS SOP. DNA from each sample was pooled into equimolar proportions and sequenced on an Illumina MiSeq platform (PE250) at the University of California, San Diego IGM Genomics Center.

CutAdapt v1.8.1^21^ (cutadapt -a ^CCTCCGCTTATTGATATGCTTAART…AGWGATCCRTTGYYRAAAGTT – discard-untrimmed – minimum-length 50 -o trimR2_001.fastq.gz R2_001.fastq.gz) was used to trim amplicon reads of region-specific primer sequences and to discard short reads and reads lacking ITS target primer sequences. Species-level Operational taxonomic units (OTUs), clustered at 97% identity, were generated *de novo* from the adapter-trimmed reads using J. Craig Venter Institute’s (JCVI’s) pipeline adaptation of UPARSE (https://github.com/JCVenterInstitute/Uparse_16S_pipeline).^22–24^ Briefly, trimmed R2 sequence reads (from ITS4-fun) were used as input. Sequences of low-quality were discarded and the remaining reads dereplicated prior to determination of abundances. Chimera filtering of the sequences was completed during clustering by the cluster_otus command within the UPARSE-OTU algorithm of USEARCH v8.1 (https://drive5.com/usearch/manual8.1/cmd_cluster_otus.html) while taxonomy was assigned to the OTUs with mothur v 1.36.1^25^ using a customized subset of the UNITE fungal ITS database^26^ as the reference (described below). OTUs and corresponding taxonomy assignment tables were generated and used in subsequent analyses. Downstream analyses (including principal coordinate analyses (PCoA) and predictive performance analyses of fungal markers for detecting ALD vs NAFLD based on relative abundance of fungal populations) were performed using the R statistical platform, as detailed below.^27^

A custom ITS database was generated from the sh_refs_qiime_ver8_97_s_all_04.02.2020 version of the UNITE database that contained both full-length and partial matches to the ITS2 region at least 50 bp in length and only contained taxa known to be host-associated. This was accomplished by first extracting host-associated fungal taxa by selecting genus names matching those in the THF database v1.6.1.^28^ Full and partial sequences at least 50 bp in length matching the ITS2 region were extracted by running the “host-associated” subset of the UNITE database through ITSx v1.1.2^29^ using the command (ITSx -i sh_refs_qiime_ver8_97_s_all_04.02.2020.THF.fasta -o UNITE_THFdb – cpu 16 –multi_thread T – positions T – not_found T – detailed_results T – partial 49 –save_regions ITS2 –table T). Non-fungal populations detected by ITS2 primers were excluded from final figures. Only fungal populations that were detected in at least one sample in the ALD and the NALFD cohorts respectively were included in the analysis. Similarly, OTUs with less than 10 sequences in the study population were removed.

### Data availability

Raw sequences from ITS2 gene sequencing were registered at NCBI under BioProjects PRJNA698272 (NAFLD cohort) and PRJNA703732 (ALD cohort).

### Statistics

Two groups with continuous outcomes were compared using the Wilcoxon-Whitney-Mann rank-sum test. Three or more groups with continuous outcomes were compared using the Kruskal–Wallis test; if the Kruskal–Wallis test was statistically significant, a pairwise Wilcoxon-Whitney-Mann rank-sum test was performed with Holm correction. Results with continuous outcomes are expressed as median, and upper and lower quartiles in brackets, if not stated otherwise. Categorical variables were compared using the Pearson’s Chi-squared test and results are expressed as number and percentage, if not stated otherwise. All statistical tests were two-sided. A *p* value equal to or less than 0.05 was considered statistically significant, uncorrected for two groups and corrected after Holm adjustment for multiplicity for three or more groups. The fungal sequence reads were normalized to obtain the proportional, relative abundance of each fungus in each patient for further statistical analysis. Fungal diversity markers Shannon index and inverse Simpson index were calculated using the “phyloseq” package in R.^30^ To calculate and visualize ß-diversity, we used principal coordinate analyses (PCoA) based on the Jaccard and Bray-Curtis dissimilarity matrices and *p* values were determined by nonparametric multivariate analysis of variance (MANOVA). Linear discriminant analysis (LDA) effect size (LEfSe) was used to identify fungal genera and species whose relative abundances differed significantly by at least 2.0 on the logarithmic LDA score between groups.^31^ Area under the curve (AUC), best threshold to maximize the Youden index, sensitivity, specificity, accuracy, positive predictive value, negative predictive value, and *p* value between two AUCs of receiver operating characteristic (ROC) curves per DeLong method were calculated using the pROC library in R; and to identify fungal genera and species with the highest feature importance for detecting ALD cohorts vs NAFLD cohorts, the mean decrease accuracy was calculated with the randomForest library in R, as described.^32,33^ Statistical analysis was performed using R statistical software, R version 1.3.1093 for Mac, 2020 the R Foundation for Statistical Computing.

## Results

### Study population with alcohol-associated liver disease (ALD) and nonalcoholic fatty liver disease (NAFLD)

The study population consisted of 58 patients with ALD, 78 patients with NAFLD, and 34 control subjects (Table 1). Both liver disease groups had significantly higher age and body mass index (BMI) medians than controls, with the NAFLD patients having a significantly higher age and BMI median than the ALD patients (NAFLD 55.6 y vs ALD 43.5 y vs controls 33.5 y as well as NAFLD 30.1 kg/m^2^ vs ALD 24.2 kg/m^2^ vs controls 21.1 kg/m^2^). The liver disease markers aspartate aminotransferase (AST), alanine aminotransferase (ALT), gamma-glutamyltransferase (GGT), alkaline phosphatase (AP), total bilirubin levels and the steatosis marker controlled attenuation parameter (CAP) and liver stiffness per FibroScan were higher in the ALD and NAFLD cohorts compared with controls, respectively (all comparisons were significant, except bilirubin and liver stiffness only had a trend for control vs ALD). Of all liver laboratory and imaging markers, only AST and albumin were significantly higher in the ALD cohort vs the NAFLD cohort (53.5 IU/L vs 35.0 IU/L and 4.6 g/L vs 4.4 g/L, respectively) (Table 1).

**Table:** *post-hoc *p* values.

	Control vs ALD	Control vs NAFLD	ALD vs NAFLD
Age	**0.004**	**<0.001**	**<0.001**
BMI	**0.002**	**<0.001**	**<0.001**
AST	**<0.001**	**<0.001**	**0.016**
ALT	**<0.001**	**<0.001**	0.462
GGT	**<0.001**	**<0.001**	0.077
AP	**<0.001**	**<0.001**	0.474
Bilirubin	0.098	**0.029**	0.340
Albumin	0.057	0.638	**0.005**
CAP [dB/m]	**<0.001**	**<0.001**	0.210
Stiffness [kPa]	0.070	**0.004**	0.070

**Table 1.** Baseline demographic and laboratory data of the study population.

	n	Control (*n* = 34)	Alcohol-associated liver disease (*n* = 58)	Nonalcoholic fatty liver disease (*n* = 78)	*p* value
Gender [male], n [%]	170	20 (58.8%)	40 (69.0%)	38 (48.7%)	0.061
Age [years]	170	33.5 [30.6;44.9]	43.5 [36.2;53.0]	55.6 [43.3;63.4]	0.**001**
BMI [kg/m^2^	170	21.1 [20.0;24.1]	24.2 [22.0;27.5]	30.1 [27.5;33.5]	**<0.001**
AST [IU/L]	160	19.0 [16.0;25.0]	53.5 [29.0;101]	35.0 [28.0;52.0]	**<0.001**
ALT [IU/L]	160	13.0 [9.00;15.0]	46.5 [24.2;87.5]	48.0 [33.0;78.0]	**<0.001**
GGT [IU/L]	160	16.0 [11.0;20.4]	108 [42.0;289]	72.0 [46.0;125]	**<0.001**
AP [IU/L]	158	55.5 [49.6;62.1]	75.0 [60.0;88.0]	75.0 [65.0;94.0]	0.**001**
Bilirubin [mg/dL]	158	0.30 [0.15;0.58]	0.50 [0.30;0.60]	0.50 [0.40;0.80]	0.**029**
Albumin [g/dL]	157	4.40 [4.40;4.50]	4.60 [4.30;4.93]	4.40 [4.20;4.60]	0.**004**
INR	148	1.00 [1.00;1.00]	0.98 [0.91;1.03]	1.00 [0.90;1.00]	0.599
Creatinine [mg/dL]	159	0.81 [0.71;1.00]	0.81 [0.72;0.87]	0.85 [0.71;1.02]	0.378
Platelets [10^9^/L]	150	240 [230;264]	231 [171;277]	217 [181;278]	0.496
CAP [dB/m]	99	195 [184;200]	310 [268;330]	280 [261;314]	**<0.001**
Stiffness [kPa]	146	4.60 [4.00;5.35]	5.50 [4.12;6.68]	6.20 [4.80;13.0]	0.**003**

Values are presented as median and upper and lower quartiles in brackets. The number of subjects for which data were available is indicated in the first column. Continuous variables were compared using the Wilcoxon-Whitney-Mann rank-sum test. Categorical variables were compared using the Pearson’s Chi-squared test. Bold font indicates statistical significance (*p* < 0.05). ALT, alanine aminotransferase; AP, alkaline phosphatase; AST, aspartate aminotransferase; BMI, body mass index; CAP, controlled attenuation parameter; GGT, gamma-glutamyltransferase; INR, international normalized ratio.

### The fungal microbiome is different in patients with ALD from NAFLD patients

**Figure 1.**
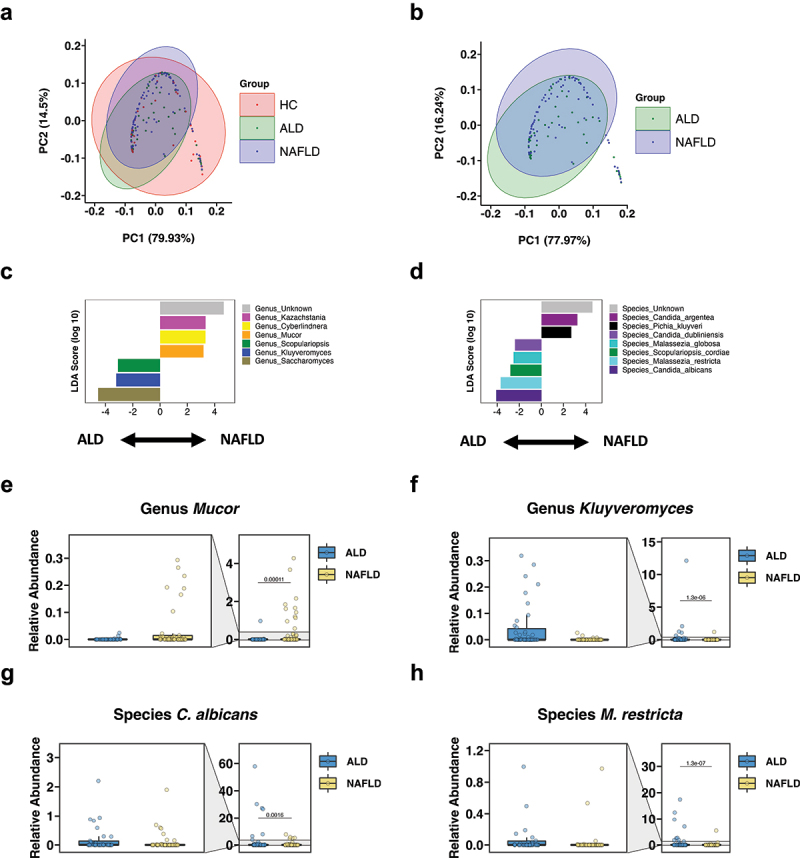
The intestinal fungal microbiome differs significantly between patients with ALD and NAFLD.

The intestinal mycobiome of controls from the ALD cohort was not significantly different from controls from the NAFLD cohort per principal coordinate analysis (PCoA, *n* = 18 vs *n* = 16, *p* = 0.116, not shown). We therefore combined both control groups (*n* = 34, Table 1). When comparing all 3 groups, there was a significant difference per nonparametric multivariate analysis of variance (MANOVA) using the Jaccard index (*p* = 0.029) (Figure 1a). The *p* value was 0.059 per Bray-Curtis dissimilarity. The fungal microbiome was significantly different between the ALD and NAFLD cohorts per Jaccard index (*p* = 0.026) (Figure 1b). The *p* value between ALD and NAFLD was 0.068 per Bray-Curtis dissimilarity. Furthermore, alpha diversity markers were different between the groups: The *p* value per Kruskal–Wallis test was 0.065 between the three groups for the Shannon Index. The Shannon Index was lower in the ALD cohort vs the NAFLD cohort (unadjusted *p* = 0.036, adjusted *p* = 0.11) (Supplementary Figure S1a). The inverse Simpson Index was significantly decreased in the ALD cohort vs the NAFLD cohort (adjusted *p* = 0.048) (Supplementary Figure S1b).

(a) Principal coordinate analysis (PCoA) of mycobiome in ALD patients (*n* = 58), NAFLD patients (*n* = 78), and controls (*n* = 34). (b) PCoA of mycobiome in ALD patients (*n* = 58) and NAFLD patients (*n* = 78). (c-d) Linear discriminant analysis (LDA) of (c) genera and (d) species of ALD patients vs NAFLD patients. (E-H) Relative abundance of genera (e) *Mucor*, (f) *Kluyveromyces*, and of species (g) *Candida albicans* and (h) *Malassezia restricta*. A *p* value of equal or less than 0.05 was considered as statistically significant.

We then performed linear discriminant analysis (LDA) effect size (or LEfSe)^31^ to identify which fungal genera and species were significantly different between the ALD and NAFLD cohorts. The genera *Saccharomyces*, *Kluyveromyces*, and *Scopulariopsis* were significantly increased in patients with ALD, whereas unidentified genera, *Kazachstania*, *Cyberlindnera*, and *Mucor* were significantly increased in the NAFLD cohort (Figure 1c). Similarly, the ALD group had significantly increased fungal species including *Candida albicans* (*C. albicans*), *Malassezia restricta* (*M. restricta*), *Scopulariopsis cordiae* (*S. cordiae*), *M. globosa*, and *C. dubliniensis*, while subjects with NAFLD had increased unknown species, *C. argentea*, and *Pichia kluyveri* (Figure 1d). Likewise, the relative abundance of the genus *Mucor* was low and that of *Kluyveromyces* elevated in the ALD group compared with the NAFLD group (Figure 1e–f). The relative abundances of the species *C. albicans* and *M. restricta* were significantly increased in the ALD cohort in relation to NAFLD (Figure 1g–h).

### A fungal signature differentiates ALD from NAFLD

**Figure 2.**
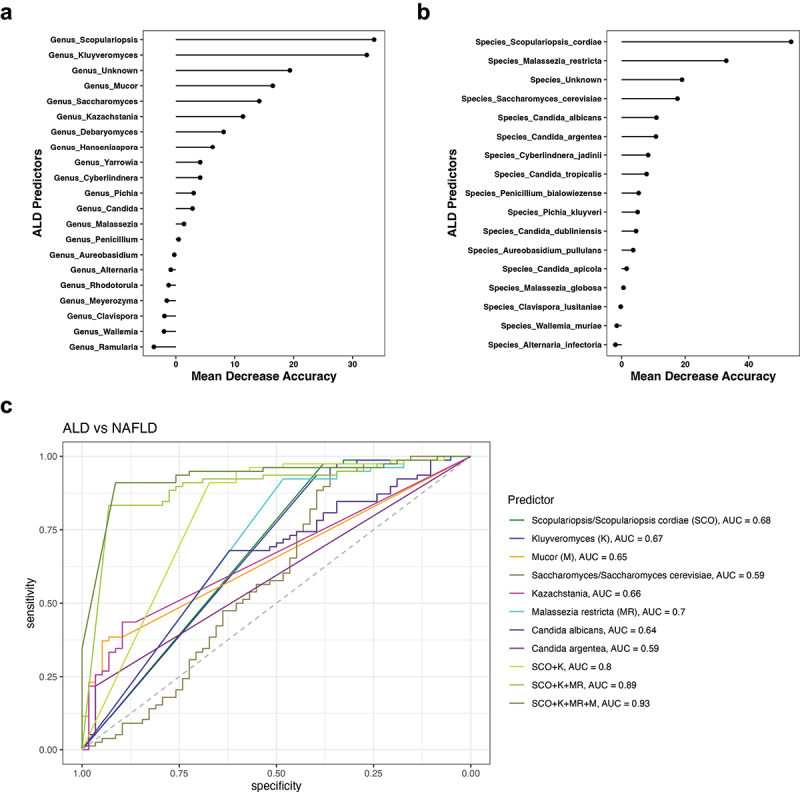
A fungal signature differentiates ALD from NAFLD.

To identify fungal genera and species with the highest feature importance for detecting ALD vs NAFLD, we determined the mean decrease accuracy per random forest analysis, as described before.^32,33^ Among the top genera differentiating ALD from NAFLD were *Scopularopsis*, *Kluyveromyces*, unidentified genera, *Mucor*, *Saccharomyces*, and *Kazachstania* (Figure 2a). Among the fungal species with the highest feature importance for detecting ALD vs NAFLD were *S. cordiae*, *M. restricta*, unknown species, *S. cerevisiae*, and *C. albicans* (Figure 2b). In a second step, we determined their discriminative value for ALD vs NAFLD (Figure 2c). The highest area under the curve (AUC) value for single fungal predictors were 0.70 for *M. restricta*, 0.68 for *Scopulariopsis* and *S. cordiae*, and 0.67 for *Kluveromyces* (Figure 2c). We then compared various combinations of the fungal markers with the highest AUC values in order to maximize the discriminative ability for ALD vs NAFLD. We identified the fungal signature consisting of *Scopulariopsis*, *Kluyveromyces*, *M. restricta*, and *Mucor* to have the highest discriminative ability with an excellent AUC of 0.93, which was significantly better per DeLong test than the AUC of 0.89 for the fungal signature comprising *Scopulariopsis*, *Kluyveromyces*, and *M. restricta*, *p* = 0.038 (Figure 2c, Table 2). The sensitivity, specificity, accuracy, positive, and negative predictive value (PPV and NPV) for this fungal signature consisting of *Scopulariopsis*, *Kluyveromyces*, *M. restricta*, and *Mucor* were 0.91, 0.91, 0.91, 0.88, and 0.93, respectively (Table 2).

(a-b) Mean decrease accuracy by random forest analysis was quantitated for (a) fungal genera and (b) species to determine their respective feature importance for detecting ALD vs NAFLD. (c) ROC curves of fungal genera and species for detecting ALD vs NAFLD (ALD patients, *n* = 58; NAFLD patients, *n* = 78). ALD, alcohol-associated liver disease; AUC, area under the curve; K, *Kluyveromyces*; M, *Mucor*; MR, *Malassezia restricta*; NAFLD, nonalcoholic fatty liver disease; ROC, receiver operating characteristic; SCO, Scopulariopsis/*S. cordiae*.

**Table 2.** ALD vs NAFLD predictors.

Marker	AUC	Youden	Threshold	Sens	Spec	Acc	PPV	NPV	*p* value DeLong’s test
*Scopulariopsis/S. cordiae (SCO)*	**0.68**	0.35	>0.0020%	0.38	0.97	0.72	0.92	0.68	
*Kluyveromyces (K)*	**0.67**	0.33	>0.0022%	0.40	0.94	0.71	0.82	0.68	
*Mucor (M)*	**0.65**	0.32	<0.0031%	0.95	0.37	0.62	0.53	0.91	
*Saccharomyces/S. cerevisiae*	**0.59**	0.30	>91.38%	0.36	0.94	0.69	0.81	0.66	
*Kazachstania*	**0.66**	0.33	<0.0024%	0.90	0.44	0.63	0.54	0.85	
*M. restricta (MR)*	**0.70**	0.41	>0.00026%	0.48	0.92	0.74	0.82	0.71	
*C. albicans*	**0.64**	0.30	>0.00026%	0.62	0.68	0.65	0.59	0.71	
*C. argentea*	**0.59**	0.18	<0.00001%	0.97	0.22	0.54	0.48	0.90	
*SCO+K*	**0.80**	0.58	*SCO* > 0.0020%, *K* > 0.0022%	0.67	0.91	0.81	0.85	0.79	**<0.001 vs *SCO***
*SCO+K+MR*	**0.89**	0.76	*SCO* > 0.0020%, *K* > 0.0022%, *MR* > 0.00026%	0.93	0.83	0.88	0.81	0.94	0.**007 vs *SCO*+*K***
*SCO+K+MR+M*	**0.93**	0.82	*SCO >0.0020%, K > 0.0022%*, MR * > 0.00026%, M* < 0.0031%	0.91	0.91	0.91	0.88	0.93	0.**038** **vs *SCO*+*K*+*MR***

The best threshold was determined to maximize the Youden index (= sensitivity + specificity − 1) for each marker. *N* = 136. Acc, accuracy; AUC, area under the curve; C., *Candida*; K, *Kluyveromyces*; M, *Mucor*; MR, *Malassezia restricta*; NPV, negative predictive value; PPV, positive predictive value; *S. cerevisiae*, *Saccharomyces cerevisiae*; SCO, Scopulariopsis/*S. cordiae*; sens, sensitivity; spec, specificity.

### Fungal subpopulations distinguish ALD and NAFLD with no or mild fibrosis

We then stratified the ALD and NAFLD cohorts by fibrosis severity into fibrosis stages F0-F1 and F2-F4 in order to evaluate whether the fungal genera and species differ already significantly with no or mild fibrosis and whether the fungal differences are even more pronounced between ALD and NAFLD with more significant fibrosis. The demographic and laboratory data for each stratum is shown in Table 3. As expected, the BMIs were significantly higher in both F0-F1 and F2-F4 NAFLD cohorts relative to their respective ALD counterparts. The AST levels were significantly higher in the ALD F0-F1 group vs the NAFLD F0-F1 group. The AST and GGT levels were significantly higher in the ALD F2-F4 vs the NAFLD F2-F4 cohorts (Table 3).

**Table:** *post-hoc *p* values.

	ALD F0-F1 vs ALD F2-F4	NAFLD F0-F1 vs NAFLD F2-F4	ALD F0-F1 vs NAFLD F0-F1	ALD F2-F4 vs NAFLD F2-F4
Age	0.13	**0.001**	0.171	0.184
BMI	0.354	0.103	**<0.001**	**0.013**
AST	**0.008**	**<0.001**	**0.017**	**0.016**
GGT	**0.003**	0.897	1.000	**0.001**
Albumin	**0.018**	**0.011**	**0.022**	0.813
INR	**<0.001**	**0.004**	0.556	**0.043**
Platelets	0.147	0.183	0.916	0.864
Stiffness	**<0.001**	**<0.001**	0.665	0.606

**Table 3.** Baseline demographic and laboratory data of the study population stratified by fibrosis severity.

	n	ALD F0-F1 (*n* = 48)	ALD F2-F4 (*n* = 10)	NAFLD F0-F1 (*n* = 43)	NAFLD F2-F4 (*n* = 30)	*p* value
Gender [male], n [%]	131	33 (68.8%)	7 (70.0%)	22 (51.2%)	14 (46.7%)	0.154
Age [years]	131	42.0 [34.0;53.0]	50.0 [43.2;62.5]	52.3 [38.5;58.6]	60.7 [55.2;66.0]	**<0.001**
BMI [kg/m^2^	131	24.2 [21.9;26.6]	25.5 [22.3;28.4]	29.6 [27.0;31.2]	31.4 [27.5;36.6]	**<0.001**
AST [IU/L]	131	47.5 [28.8;84.8]	107 [64.5;178]	30.0 [26.5;37.5]	50.0 [33.2;63.2]	**<0.001**
ALT [IU/L]	131	39.5 [23.0;77.5]	81.0 [46.8;93.5]	44.0 [30.5;58.5]	54.0 [34.0;84.0]	0.135
GGT [IU/L]	131	90.0 [40.2;212]	415 [259;896]	83.0 [42.0;120]	70.5 [49.5;124]	0.**002**
AP [IU/L]	130	73.0 [61.0;86.0]	88.0 [59.0;135]	73.0 [61.0;93.0]	77.5 [67.2;94.5]	0.343
Bilirubin [mg/dL]	130	0.50 [0.30;0.60]	0.55 [0.40;0.70]	0.40 [0.30;0.70]	0.60 [0.40;0.90]	0.128
Albumin [g/dL]	129	4.65 [4.43;5.00]	4.40 [4.03;4.57]	4.50 [4.30;4.65]	4.30 [4.03;4.47]	**<0.001**
INR	130	0.94 [0.89;1.01]	1.11 [1.04;1.21]	1.00 [0.90;1.00]	1.00 [1.00;1.10]	**<0.001**
Creatinine [mg/dL]	131	0.81 [0.74;0.92]	0.74 [0.71;0.81]	0.85 [0.70;1.03]	0.84 [0.70;0.98]	0.361
Platelets [10^9^/L]	130	240 [188;284]	169 [136;230]	250 [186;287]	208 [146;250]	0.**028**
CAP [dB/m]	84	308 [265;326]	328 [295;347]	279 [249;309]	291 [269;325]	0.321
Stiffness [kPa]	131	5.05 [3.98;6.12]	17.2 [12.7;24.4]	4.80 [4.40;5.55]	13.7 [11.0;17.5]	**<0.001**

Values are presented as median and upper and lower quartiles in brackets. The number of subjects for which data were available is indicated in the first column. Continuous variables were compared using the Wilcoxon-Whitney-Mann rank-sum test. Categorical variables were compared using the Pearson’s Chi-squared test. Bold font indicates statistical significance (*p* < 0.05). ALT, alanine aminotransferase; AP, alkaline phosphatase; AST, aspartate aminotransferase; BMI, body mass index; CAP, controlled attenuation parameter; GGT, gamma-glutamyltransferase; INR, international normalized ratio.

**Figure 3.**
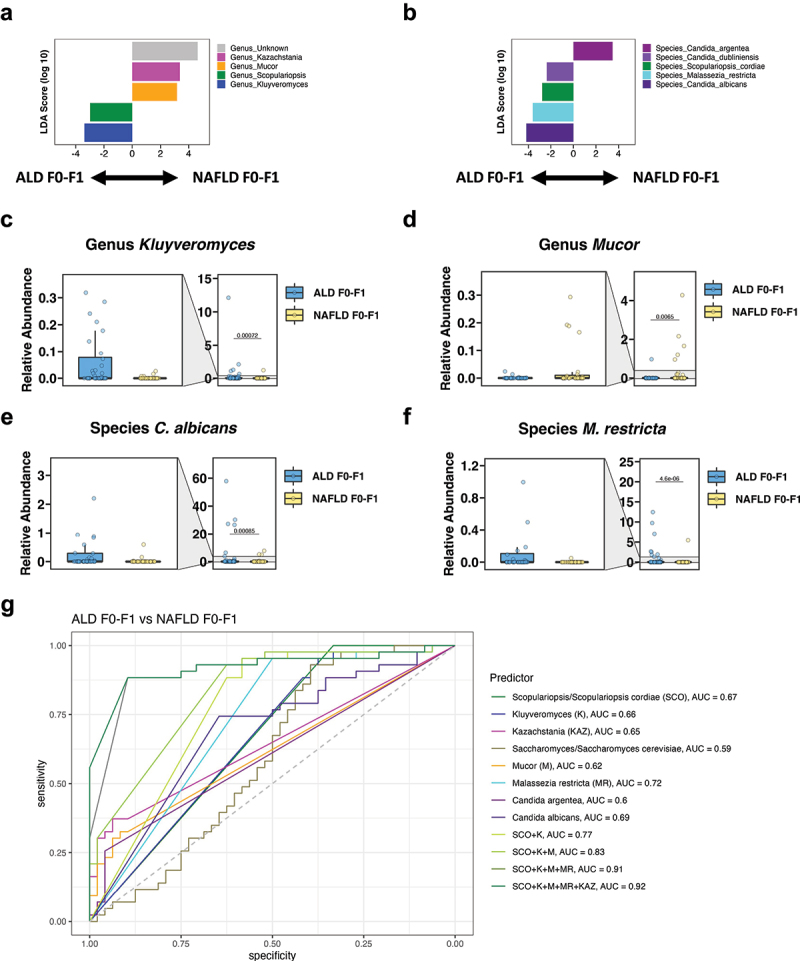
Fungal subpopulations distinguish ALD and NAFLD with no or mild fibrosis.

When comparing the cohorts with no or mild fibrosis, the ALD F0-F1 had significantly increased fungal genera *Kluyveromyces* and *Scopulariopsis* and significantly decreased unknown genera, *Kazachstania*, and *Mucor* compared with NAFLD F0-F1 per LEfSe (Figure 3a). Additionally, *C. albicans*, *C. dubliniensis*, *M. restricta*, and *S. cordiae* were significantly enriched and *C. argentea* was significantly reduced in the ALD F0-F1 vs the NAFLD F0-F1 groups per LEfSe (Figure 3b). Consistent with these results, the fecal relative abundance of *Kluyveromyces*, *C. albicans*, and *M. restricta* was significantly increased in the ALD F0-F1 cohort, whereas the fecal relative abundance of *Mucor* was significantly decreased in the ALD F0-F1 cohort compared with the NAFLD F0-F1 cohort (Figure 3c–f). The fungal genera and species with the highest feature importance for detecting ALD F0-F1 vs NAFLD F0-F1 as expressed as the highest mean decrease accuracy per random forest analysis were *Scopulariopsis*, *Kluyveromyces*, *Kazachstania*, *Saccharomyces*, and *Mucor* as well as *S. cordiae*, *M. restricta*, *C. argentea*, *S. cerevisiae*, and *C. albicans* (Supplementary Figure S2a-b). Finally, we determined their discriminative value for ALD F0-F1 vs NAFLD F0-F1 (Figure 3g). The highest AUC for single fungal predictors were 0.72 for *M. restricta*, 0.69 for *C. albicans*, and 0.67 for *Scopulariopsis* and *S. cordiae*. The fungal signature with the highest AUC of 0.92 to discriminate ALD F0-F1 from NAFLD F0-F1 comprised *Scopulariopsis*, *Kluyveromyces*, *Mucor*, *M. restricta*, and *Kazachstania* (Figure 3g).

(a-b) Linear discriminant analysis (LDA) of (a) genera and (b) species of ALD F0-F1 vs NAFLD F0-F1. (c-f) Relative abundance of genera (c) *Kluyveromyces*, (d) *Mucor*, and of species (e) *Candida albicans* and (f) *Malassezia restricta*. (g) ROC curves of fungal genera and species for detecting ALD F0-F1 vs NAFLD F0-F1 (ALD F0-F1 patients, *n* = 48; NAFLD F0-F1 patients, *n* = 43). A *p* value of equal or less than 0.05 was considered as statistically significant. ALD, alcohol-associated liver disease; AUC, area under the curve; K, *Kluyveromyces*; KAZ, *Kazachstania*; M, *Mucor*; MR, *Malassezia restricta*; NAFLD, nonalcoholic fatty liver disease; ROC, receiver operating characteristic; SCO, Scopulariopsis/*S. cordiae*.

### A fungal signature differentiates ALD from NAFLD with significant fibrosis

**Figure 4.**
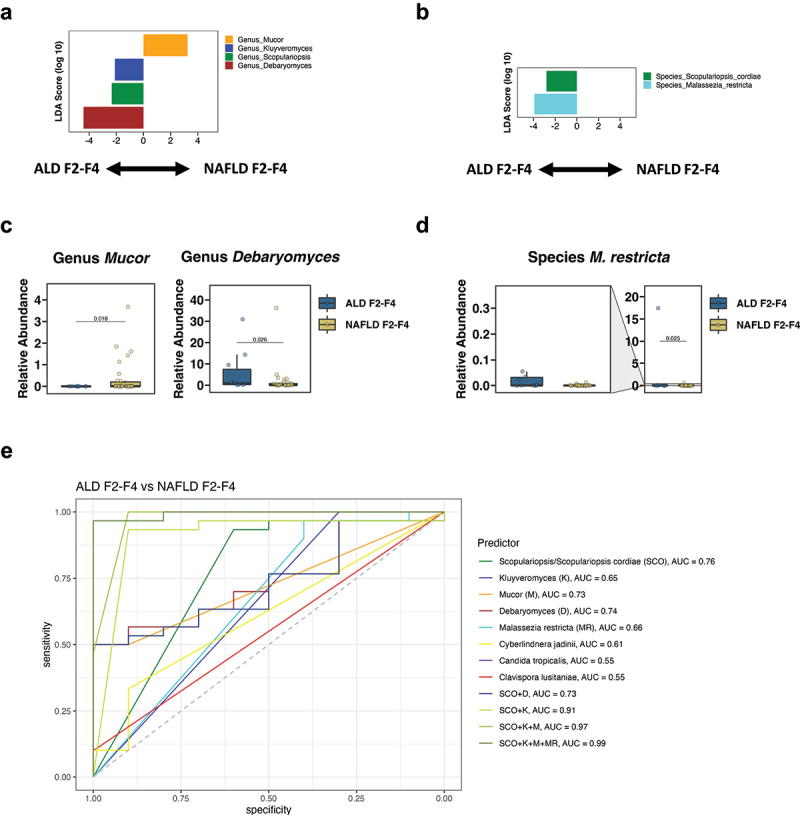
A fungal signature differentiates ALD from NAFLD with significant fibrosis.

We next compared both liver disease cohorts with significant fibrosis. The ALD F2-F4 had significantly enriched fungal genera *Debaryomyces*, *Scopulariopsis*, and *Kluyveromyces* and significantly decreased *Mucor* compared with NAFLD F2-F4 per LEfSe (Figure 4a). Moreover, *M. restricta*, and *S. cordiae* were significantly increased in the ALD F2-F4 vs the NAFLD F2-F4 groups per LEfSe (Figure 4b). Likewise, the fecal relative abundances of *Debaryomyces* and *M. restricta* were significantly increased in the ALD F2-F4 cohort, whereas the fecal relative abundance of *Mucor* was significantly decreased in the ALD F2-F4 cohort compared with the NAFLD F2-F4 cohort (Figure 4c–d). The fungal genera and species with the highest feature importance for identifying ALD F2-F4 vs NAFLD F2-F4 per mean decrease accuracy were *Scopulariopsis*, *Kluyveromyces*, *Mucor*, and *Debaryomyces* as well as *S. cordiae*, *M. restricta*, and *Cyberlindera jadinii* (Supplementary Figure S2c-d). Lastly, we determined their discriminative value for ALD F2-F4 vs NAFLD F2-F4 (Figure 4e). The highest AUC for single fungal predictors were 0.76 for *Scopulariopsis* and *S. cordiae*, 0.74 for *Debaryomyces*, and 0.73 for *Mucor*. The fungal signature with the highest AUC of 0.99 to identify ALD F2-F4 vs NAFLD F2-F4 included *Scopulariopsis*, *Kluyveromyces*, *Mucor*, and *M. restricta* (Figure 4e).

(a-b) Linear discriminant analysis (LDA) of (a) genera and (b) species of ALD F2-F4 vs NAFLD F2-F4. (c-d) Relative abundance of genera (c) *Mucor* and *Debaryomyces*, and of species (d) *Malassezia restricta*. (e) ROC curves of fungal genera and species for detecting ALD F2-F4 vs NAFLD F2-F4 (ALD F2-F4 patients, *n* = 10; NAFLD F2-F4 patients, *n* = 30). A *p* value of equal or less than 0.05 was considered as statistically significant. ALD, alcohol-associated liver disease; AUC, area under the curve; K, *Kluyveromyces*; M, *Mucor*; MR, *Malassezia restricta*; NAFLD, nonalcoholic fatty liver disease; ROC, receiver operating characteristic; SCO, Scopulariopsis/*S. cordiae*.

## Discussion

Here, we report that the fungal microbiota differs significantly between ALD and NAFLD. Importantly, a specific fungal signature comprising *Scopulariopsis*, *Kluyveromyces*, *Mucor*, and *M. restricta* (with or without *Kazachstania*) can differentiate ALD from NAFLD in general as well as stratified by fibrosis severity with an excellent discriminative ability (AUC > 0.9). A bacterial microbiome signature for the diagnosis of advanced fibrosis and cirrhosis has been identified for NAFLD previously.^8,9^ However, this current study is the first to report a fungal signature in liver disease.

We have previously investigated the fungal microbiome in ALD^11,12^ and NAFLD^13^ separately, and found that the relative abundance of *C. albicans* correlated with disease severity in both ALD and NAFLD. We found that fecal proportions of *C. albicans*, *Mucor* species, *Pichia barkeri*, and *Cyberlindnera jadinii* are significantly higher in patients with nonalcoholic steatohepatitis (NASH) vs nonalcoholic fatty liver and also higher in patients with NAFLD and fibrosis stages F2-F4 vs patients with NAFLD and fibrosis stages F0-F1.^13^ Further, plasma anti-*C. albicans* immunoglobulin G levels are significantly higher in patients with NAFLD and advanced fibrosis vs patients with NAFLD and no, mild, or moderate fibrosis only or healthy controls.^13^ Of note, we trialed a non-absorbable antifungal (amphotericin B) orally in a 20-week long western diet experiment in a prior study, and found that amphotericin B improves liver cell injury per ALT levels, hepatic triglycerides, liver inflammation and fibrosis in experimental diet-induced steatohepatitis in mice.^13^ Antifungal therapy could hence represent an attractive new therapy in NAFLD. Similarly, the relative abundance of *C. albicans* correlates with disease severity in ALD and is especially high in alcohol-associated hepatitis, and the level of anti–*S. cerevisiae* IgG antibodies (ASCA) – that *C. albicans* is an important immunogen for – predicts survival in alcohol-associated hepatitis.^10–12^ Likewise, use of antifungals improves experimental ethanol-induced steatohepatitis as well.^10^

In the current study, *C. albicans* was increased in the entire ALD and the ALD F0-F1 cohorts compared with the entire NAFLD and the NAFLD F0-F1 cohorts, respectively, but we did not find a significant difference between both F2-F4 cohorts. This finding could be secondary to a type II error given a lower number of patients with ALD F2-F4 (*n* = 10).^34^ Of note, *C. albicans* did not significantly improve the discriminative ability to distinguish ALD from NAFLD, nor when stratified by fibrosis severity.

Interestingly, the fungal signature can differentiate ALD from NAFLD already in early stages when no or only mild fibrosis is present. This means that fungal dysbiosis – or an imbalance of beneficial and potentially pathogenic microbes/fungi^19,35^ – is already present in early disease stages and specific for ALD and NAFLD. It is well known that diet shapes the bacterial^36^ and fungal microbiome.^37^ Fecal proportions of *C. albicans* are significantly higher in patients with ALD compared with control subjects.^11,12^ Intriguingly, only 2 weeks of alcohol abstinence significantly decrease the relative abundance of *C. albicans* but also that of *Kluyveromyces* and *M. restricta* in patients with alcohol use disorder,^12^ indicating that alcohol as part of the diet molds the fungal microbiota. Similarly, vegetarian or animal-based diets affect the fungal microbiome structure as well.^38^ However, although diet impacts the microbiota, the identified fungal signature consisting of *Scopulariopsis*, *Kluyveromyces*, *Mucor*, and *M. restricta* actually has a significantly better discriminative ability for ALD F2-F4 vs NAFLD F2-F4 than for ALD F0-F1 vs NAFLD F0-F1 (AUC = 0.99 vs AUC = 0.91), indicating that the disease-specific fungal structure is more solidified in more advanced disease stages than in earlier stages.

Previously, bacterial signatures have been identified for NAFLD and ALD. Advanced fibrosis in NAFLD has been associated with significantly lower proportions of Firmicutes, *Ruminococcus obeum*, *Eubacterium rectale*, and *Faecalibacterium prausnitzii* vs mild-to-moderate fibrosis, whereas Proteobacteria were more numerous.^8^ NAFLD-cirrhosis is associated with an increased fecal relative abundance of Enterobacteriaceae, *Veillonella parvula*, *Veillonella atypica*, *Ruminococcus gnavus*, *Clostridium bolteae* and *Acidaminococcus* sp. D21 and a reduced relative abundance of *Catenibacterium* compared with controls, and lower Peptostreptococcaceae than patients with NAFLD but without advanced fibrosis.^9,39^ On the other hand, common fecal signatures of alcohol-associated hepatitis include increased Bacilli, Lactobacillales, *Veillonella*, and decreased *Bacteroides*, *Akkermansia*, *Eubacterium*, *Oscillibacter* and Clostridiales compared with healthy controls.^40,41^ Patients with alcohol use disorder also have decreased *Akkermansia* compared with controls but increased *Bacteroides*.^42^ *Enterococcus faecalis* and presence of its secreted protein cytolysin predict disease severity and mortality in alcohol-associated hepatitis.^43^

Further, it is important to note that the gut microbiota plays a central role in maintaining liver homeostasis, but it can also function as a reservoir of pathobionts and their products that can contribute to the pathogenesis of ALD and NAFLD. There is a bidirectional crosstalk between the intestine and the liver, which involves multiple molecules and products including nutrients, microbial antigens, metabolites, and bile acids, regulating metabolism and immune responses, thereby controlling gastrointestinal health and liver diseases.^44–46^

In conclusion, this is the first study to identify a fungal signature to differentiate two fatty liver diseases from each other, specifically ALD from NAFLD. This might have clinical utility in unclear cases, as liver histology is oftentimes very similar in both conditions, and might hence help modify treatment approaches. However, larger clinical studies are required to validate this fungal signature in other populations of ALD and NAFLD.

## Abbreviations

ALD: alcohol-associated liver disease
ALT: alanine aminotransferase
AP: alkaline phosphatase
AST: aspartate aminotransferase
AUC: area under the curve
BMI: body mass index
CAP: controlled attenuation parameter
GGT: gamma-glutamyltransferase
INR: international normalized ratio
ITS: Internal transcribed spacer 2
K: *Kluyveromyces*
KAZ: *Kazachstania*
LDA: Linear discriminant analysis
LEfSe: LDA effect size
M: *Mucor*
MANOVA: multivariate analysis of variance
MR: *Malassezia restricta*
NAFLD: nonalcoholic fatty liver disease
NPV: negative predictive value
PCoA: Principal coordinate analysis
PPV: positive predictive value
ROC: receiver operating characteristic
SCO: Scopulariopsis/*S. cordiae*.

## Supplementary Material

Fungal signature differentiates Suppl Figures_R1.docx

Fungal signature differentiates ALD from NAFLD Suppl Material_R1.docx

Fungal signature differentiates Suppl Material_R1.docx

## References

[1] Niu X, Zhu L, Xu Y, Zhang M, Hao Y, Ma L, Li Y, Xing H. Global prevalence, incidence, and outcomes of alcohol related liver diseases: a systematic review and meta-analysis. BMC Public Health. 2023;23(1):859. doi:10.1186/s12889-023-15749-x. 37170239 PMC10173666

[2] Huang DQ, Mathurin P, Cortez-Pinto H, Loomba R. Global epidemiology of alcohol-associated cirrhosis and HCC: trends, projections and risk factors. Nat Rev Gastroenterol Hepatol. 2023;20(1):37–15. doi:10.1038/s41575-022-00688-6. 36258033 PMC9579565

[3] Hartmann P, Zhang X, Loomba R, Schnabl B. Global and national prevalence of nonalcoholic fatty liver disease in adolescents: an analysis of the global burden of disease study 2019. Hepatology. 2023;78(4):1168–1181. doi:10.1097/HEP.0000000000000383. 37021791 PMC10521800

[4] Younossi ZM, Golabi P, Paik JM, Henry A, Van Dongen C, Henry L. The global epidemiology of nonalcoholic fatty liver disease (NAFLD) and nonalcoholic steatohepatitis (NASH): a systematic review. Hepatology. 2023;77(4):1335–47. doi:10.1097/HEP.0000000000000004. 36626630 PMC10026948

[5] Ginès P, Castera L, Lammert F, Graupera I, Serra-Burriel M, Allen AM, Wong VWS, Hartmann P, Thiele M, Caballeria L. et al. Population screening for liver fibrosis: toward early diagnosis and intervention for chronic liver diseases. Hepatology. 2022;75(1):219–228. doi:10.1002/hep.32163. 34537988

[6] Jayasekera D, Hartmann P. Noninvasive biomarkers in pediatric nonalcoholic fatty liver disease. World J Hepatol. 2023;15(5):609–40. doi:10.4254/wjh.v15.i5.609. 37305367 PMC10251277

[7] Hartmann P, Chu H, Duan Y, Schnabl B. Gut microbiota in liver disease: too much is harmful, nothing at all is not helpful either. Am J Physiol Gastrointest Liver Physiol. 2019;316(5):G563–G73. doi:10.1152/ajpgi.00370.2018. 30767680 PMC6580239

[8] Loomba R, Seguritan V, Li W, Long T, Klitgord N, Bhatt A, Dulai PS, Caussy C, Bettencourt R, Highlander SK. et al. Gut microbiome-based metagenomic signature for non-invasive detection of advanced fibrosis in human nonalcoholic fatty liver disease. Cell Metab. 2017;25(5):1054–62.e5. doi:10.1016/j.cmet.2017.04.001. 28467925 PMC5502730

[9] Caussy C, Tripathi A, Humphrey G, Bassirian S, Singh S, Faulkner C, Bettencourt R, Rizo E, Richards L, Xu ZZ. et al. A gut microbiome signature for cirrhosis due to nonalcoholic fatty liver disease. Nat Commun. 2019;10(1):1406. doi:10.1038/s41467-019-09455-9. 30926798 PMC6440960

[10] Yang AM, Inamine T, Hochrath K, Chen P, Wang L, Llorente C, Bluemel S, Hartmann P, Xu J, Koyama Y. et al. Intestinal fungi contribute to development of alcoholic liver disease. J Clin Invest. 2017;127(7):2829–41. doi:10.1172/JCI90562. 28530644 PMC5490775

[11] Lang S, Duan Y, Liu J, Torralba MG, Kuelbs C, Ventura-Cots M, Abraldes JG, Bosques‐Padilla F, Verna EC, Brown RS. et al. Intestinal fungal dysbiosis and systemic immune response to fungi in patients with alcoholic hepatitis. Hepatology. 2020;71(2):522–38. doi:10.1002/hep.30832. 31228214 PMC6925657

[12] Hartmann P, Lang S, Zeng S, Duan Y, Zhang X, Wang Y, Bondareva M, Kruglov A, Fouts, DE, Stärkel P. et al. Dynamic changes of the fungal microbiome in alcohol use disorder. Front Physiol. 2021;12:699253. doi:10.3389/fphys.2021.699253. 34349667 PMC8327211

[13] Demir M, Lang S, Hartmann P, Duan Y, Martin A, Miyamoto Y, Bondareva M, Zhang X, Wang Y, Kasper P. et al. The fecal mycobiome in non-alcoholic fatty liver disease. J Hepatol. 2022;76(4):788–99. doi:10.1016/j.jhep.2021.11.029. 34896404 PMC8981795

[14] Hsu CL, Lang S, Demir M, Fouts DE, Stärkel P, Schnabl B. Any alcohol use in NAFLD patients is associated with significant changes to the intestinal virome. Hepatology. 2023;77(6):2073–83. doi:10.1097/HEP.0000000000000238. 36631002 PMC10192041

[15] Maccioni L, Gao B, Leclercq S, Pirlot B, Horsmans Y, De Timary P, Leclercq I, Fouts D, Schnabl B, Stärkel P. et al. Intestinal permeability, microbial translocation, changes in duodenal and fecal microbiota, and their associations with alcoholic liver disease progression in humans. Gut Microbes. 2020;12(1):1782157. doi:10.1080/19490976.2020.1782157. 32588725 PMC7524402

[16] Maccioni L, Horsmans Y, Leclercq I, Schnabl B, Stärkel P. Serum keratin 18-M65 levels detect progressive forms of alcohol-associated liver disease in early noncirrhotic stages. Alcohol (Hanover). 2023;47(6):1079–1087. doi:10.1111/acer.15081. PMC10803128 37060262

[17] Naveau S, Lamouri K, Pourcher G, Njiké-Nakseu M, Ferretti S, Courie R, Tranchart H, Ghinoiu M, Balian A, Prévot S. et al. The diagnostic accuracy of transient elastography for the diagnosis of liver fibrosis in bariatric surgery candidates with suspected NAFLD. Obes Surg. 2014;24(10):1693–701. doi:10.1007/s11695-014-1235-9. 24841950

[18] Yang X, Chang X, Wu S, Sun X, Zhu X, Wang L, Xu Y, Yao X, Rao S, Hu X. et al. Performance of liver stiffness measurements obtained with FibroScan is affected by glucose metabolism in patients with nonalcoholic fatty liver disease. Lipids Health Dis. 2021;20(1):27. doi:10.1186/s12944-021-01453-5. 33757528 PMC7986416

[19] Hartmann P, Schnabl B. New developments in microbiome in alcohol-associated and nonalcoholic fatty liver disease. Semin Liver Dis. 2021;41(1):87–102. doi:10.1055/s-0040-1719174. 33957682 PMC8163568

[20] Taylor DL, Walters WA, Lennon NJ, Bochicchio J, Krohn A, Caporaso JG, Pennanen T. Accurate estimation of fungal diversity and abundance through improved lineage-specific primers optimized for Illumina Amplicon sequencing. Appl Environ Microb. 2016;82(24):7217–7226. doi:10.1128/AEM.02576-16. PMC5118932 27736792

[21] Martin M. Cutadapt removes adapter sequences from high-throughput sequencing reads. EMBnet J. 2011;17(1):3. doi:10.14806/ej.17.1.200.

[22] Edgar RC. UPARSE: highly accurate OTU sequences from microbial amplicon reads. Nat Methods. 2013;10(10):996–8. doi:10.1038/nmeth.2604. 23955772

[23] Freire M, Moustafa A, Harkins DM, Torralba MG, Zhang Y, Leong P, Saffery R, Bockmann M, Kuelbs C, Hughes T. et al. Longitudinal study of oral microbiome variation in twins. Sci Rep. 2020;10(1):7954. doi:10.1038/s41598-020-64747-1. 32409670 PMC7224172

[24] Singh H, Torralba MG, Moncera KJ, DiLello L, Petrini J, Nelson KE, Pieper R. Gastro-intestinal and oral microbiome signatures associated with healthy aging. Geroscience. 2019;41(6):907–921. doi:10.1007/s11357-019-00098-8. 31620923 PMC6925087

[25] Schloss PD, Westcott SL, Ryabin T, Hall JR, Hartmann M, Hollister EB, Lesniewski RA, Oakley BB, Parks DH, Robinson CJ. et al. Introducing mothur: open-source, platform-independent, community-supported software for describing and comparing microbial communities. Appl Environ Microb. 2009;75(23):7537–41. doi:10.1128/AEM.01541-09. PMC2786419 19801464

[26] Nilsson RH, Larsson KH, Taylor AFS, Bengtsson-Palme J, Jeppesen TS, Schigel D, Kennedy P, Picard K, Glöckner FO, Tedersoo L. et al. The UNITE database for molecular identification of fungi: handling dark taxa and parallel taxonomic classifications. Nucleic Acids Res. 2019;47(D1):D259–D264. doi:10.1093/nar/gky1022. 30371820 PMC6324048

[27] R Core Team. R: a language and environment for statistical computing. Vienna, Austria: R Foundation for Statistical Computing; 2018. http://www.R-project.org/.

[28] Tang J, Iliev ID, Brown J, Underhill DM, Funari VA. Mycobiome: approaches to analysis of intestinal fungi. J Immunol Methods. 2015;421:112–121. doi:10.1016/j.jim.2015.04.004. 25891793 PMC4451377

[29] Bengtsson-Palme J, Ryberg M, Hartmann M, Branco S, Wang Z, Godhe A, De Wit P, Sánchez‐García M, Ebersberger I, de Sousa F. et al. Improved software detection and extraction of ITS1 and ITS2 from ribosomal ITS sequences of fungi and other eukaryotes for analysis of environmental sequencing data. Methods Ecol Evol. 2013;4(10):914–919. doi:10.1111/2041-210X.12073.

[30] McMurdie PJ, Holmes S. Phyloseq: an R package for reproducible interactive analysis and graphics of microbiome census data. PloS ONE. 2013;8(4):e61217. doi:10.1371/journal.pone.0061217. 23630581 PMC3632530

[31] Segata N, Izard J, Waldron L, Gevers D, Miropolsky L, Garrett WS, Huttenhower C. Metagenomic biomarker discovery and explanation. Genome Biol. 2011;12(6):R60. doi:10.1186/gb-2011-12-6-r60. 21702898 PMC3218848

[32] Cabré N, Hartmann P, Llorente C, Kouno T, Wang Y, Zeng S, Kim HY, Zhang X, Kisseleva T, Iyer S. et al. IgY antibodies against cytolysin reduce ethanol-induced liver disease in mice. Hepatology. 2023;78(1):295–306. doi:10.1097/HEP.0000000000000324. 36811393 PMC10293100

[33] Hartmann P, Lang S, Schierwagen R, Klein S, Praktiknjo M, Trebicka J, Schnabl B. Fecal cytolysin does not predict disease severity in acutely decompensated cirrhosis and acute-on-chronic liver failure. Hepatobiliary Pancreat Dis Int. 2023;22(5):474–481. doi:10.1016/j.hbpd.2023.05.003. 37198098 PMC10797562

[34] Zhang X, Hartmann P. How to calculate sample size in animal and human studies. Front Med. 2023;10:1215927. doi:10.3389/fmed.2023.1215927. PMC10469945 37663663

[35] Hartmann P, Schnabl B. Risk factors for progression of and treatment options for NAFLD in children. Clin Liver Dis (Hoboken). 2018;11(1):11–5. doi:10.1002/cld.685. 29629177 PMC5881937

[36] Kaufmann B, Seyfried N, Hartmann D, Hartmann P. Probiotics, prebiotics, and synbiotics in nonalcoholic fatty liver disease and alcohol-associated liver disease. Am J Physiol Gastrointest Liver Physiol. 2023;325(1):G42–G61. doi:10.1152/ajpgi.00017.2023. 37129252 PMC10312326

[37] Hartmann P, Schnabl B. Fungal infections and the fungal microbiome in hepatobiliary disorders. J Hepatol. 2023;78:836–851. doi:10.1016/j.jhep.2022.12.006. 36565724 PMC10033447

[38] David LA, Maurice CF, Carmody RN, Gootenberg DB, Button JE, Wolfe BE, Ling AV, Devlin AS, Varma Y, Fischbach MA. et al. Diet rapidly and reproducibly alters the human gut microbiome. Nature. 2014;505(7484):559–63. doi:10.1038/nature12820. 24336217 PMC3957428

[39] Oh TG, Kim SM, Caussy C, Fu T, Guo J, Bassirian S, Singh S, Madamba EV, Bettencourt R, Richards L. et al. A Universal Gut-Microbiome-Derived Signature Predicts Cirrhosis. Cell Metab. 2020;32(5):878–88.e6. doi:10.1016/j.cmet.2020.06.005. 32610095 PMC7822714

[40] Kim SS, Eun JW, Cho HJ, Song DS, Kim CW, Kim YS, Lee SW, Kim Y-K, Yang J, Choi J. et al. Microbiome as a potential diagnostic and predictive biomarker in severe alcoholic hepatitis. Aliment Pharmacol Ther. 2021;53(4):540–51. doi:10.1111/apt.16200. 33264437

[41] Lang S, Fairfied B, Gao B, Duan Y, Zhang X, Fouts DE, Schnabl B. Changes in the fecal bacterial microbiota associated with disease severity in alcoholic hepatitis patients. Gut Microbes. 2020;12(1):1785251. doi:10.1080/19490976.2020.1785251. 32684075 PMC7524371

[42] Addolorato G, Ponziani FR, Dionisi T, Mosoni C, Vassallo GA, Sestito L, Petito V, Picca A, Marzetti E, Tarli C. et al. Gut microbiota compositional and functional fingerprint in patients with alcohol use disorder and alcohol-associated liver disease. Liver Int. 2020;40(4):878–88. doi:10.1111/liv.14383. 31951082

[43] Duan Y, Llorente C, Lang S, Brandl K, Chu H, Jiang L, White RC, Clarke TH, Nguyen K, Torralba M. et al. Bacteriophage targeting of gut bacterium attenuates alcoholic liver disease. Nature. 2019;575(7783):505–11. doi:10.1038/s41586-019-1742-x. 31723265 PMC6872939

[44] Lang S, Schnabl B. Microbiota and fatty liver disease—the known, the unknown, and the future. Cell Host & Microbe. 2020;28(2):233–244. doi:10.1016/j.chom.2020.07.007. 32791115 PMC7467841

[45] Tilg H, Adolph TE, Trauner M. Gut-liver axis: pathophysiological concepts and clinical implications. Cell Metab. 2022;34(11):1700–1718. doi:10.1016/j.cmet.2022.09.017. 36208625

[46] Bajaj JS, Khoruts A. Microbiota changes and intestinal microbiota transplantation in liver diseases and cirrhosis. J Hepatol. 2020;72(5):1003–27. doi:10.1016/j.jhep.2020.01.017. 32004593

