# Supplementary figures and images for: Fungal signature differentiates alcohol-associated liver disease from nonalcoholic fatty liver disease
Source: Gut Microbes. 2024 Feb 1;16(1):2307586. doi: 10.1080/19490976.2024.2307586 (PMC10841010; doi:10.1080/19490976.2024.2307586)

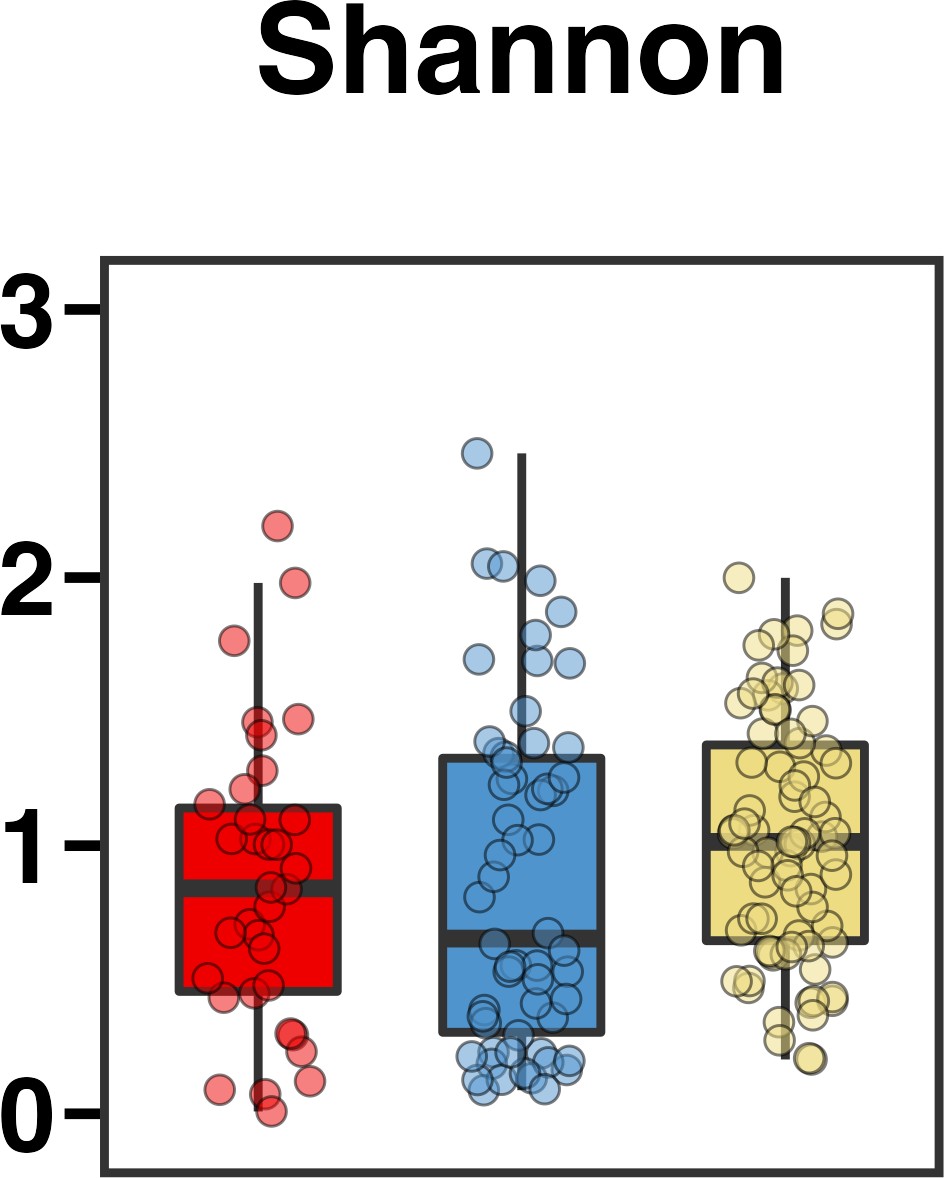


***P*=0.065**


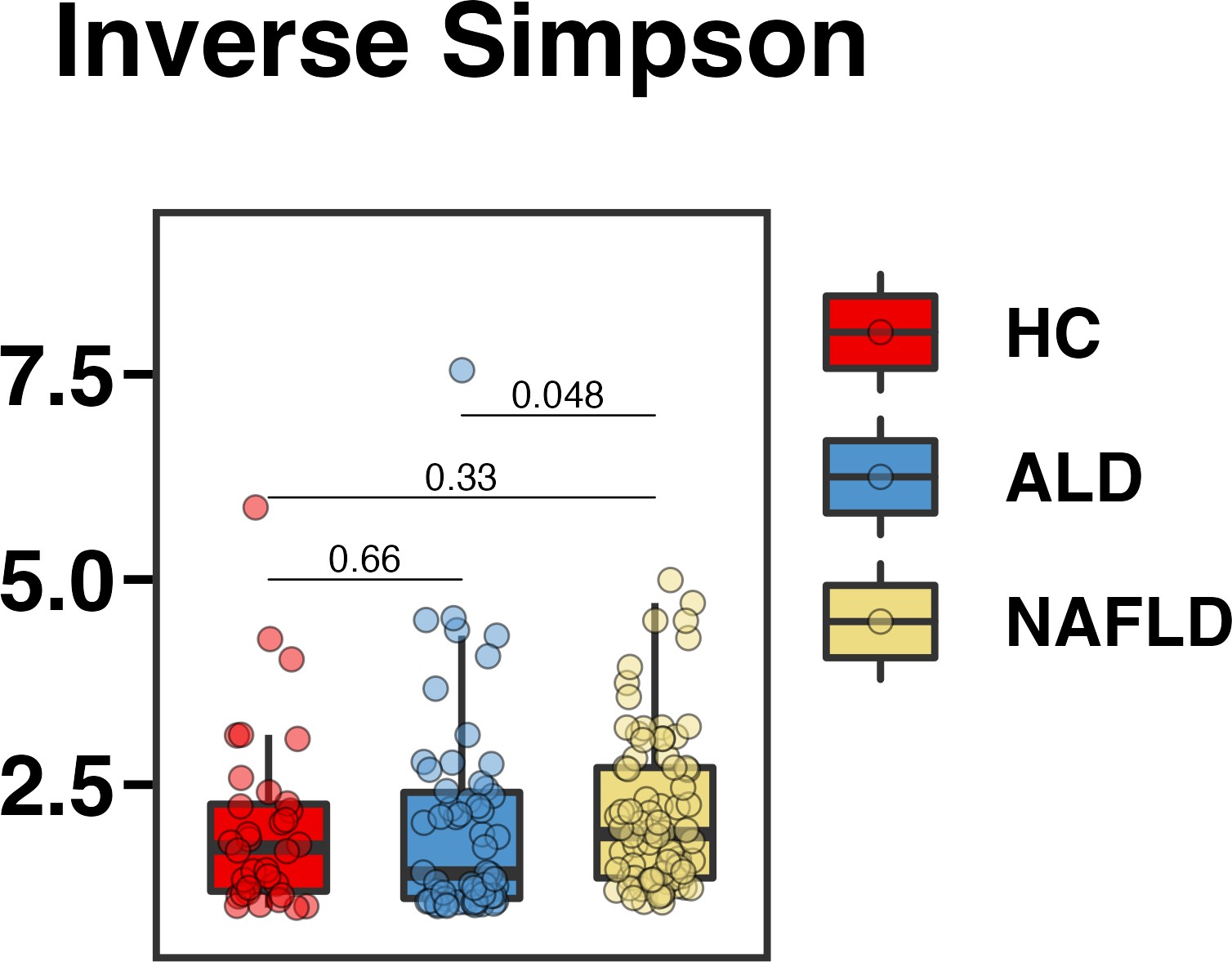


***P*=0.049**


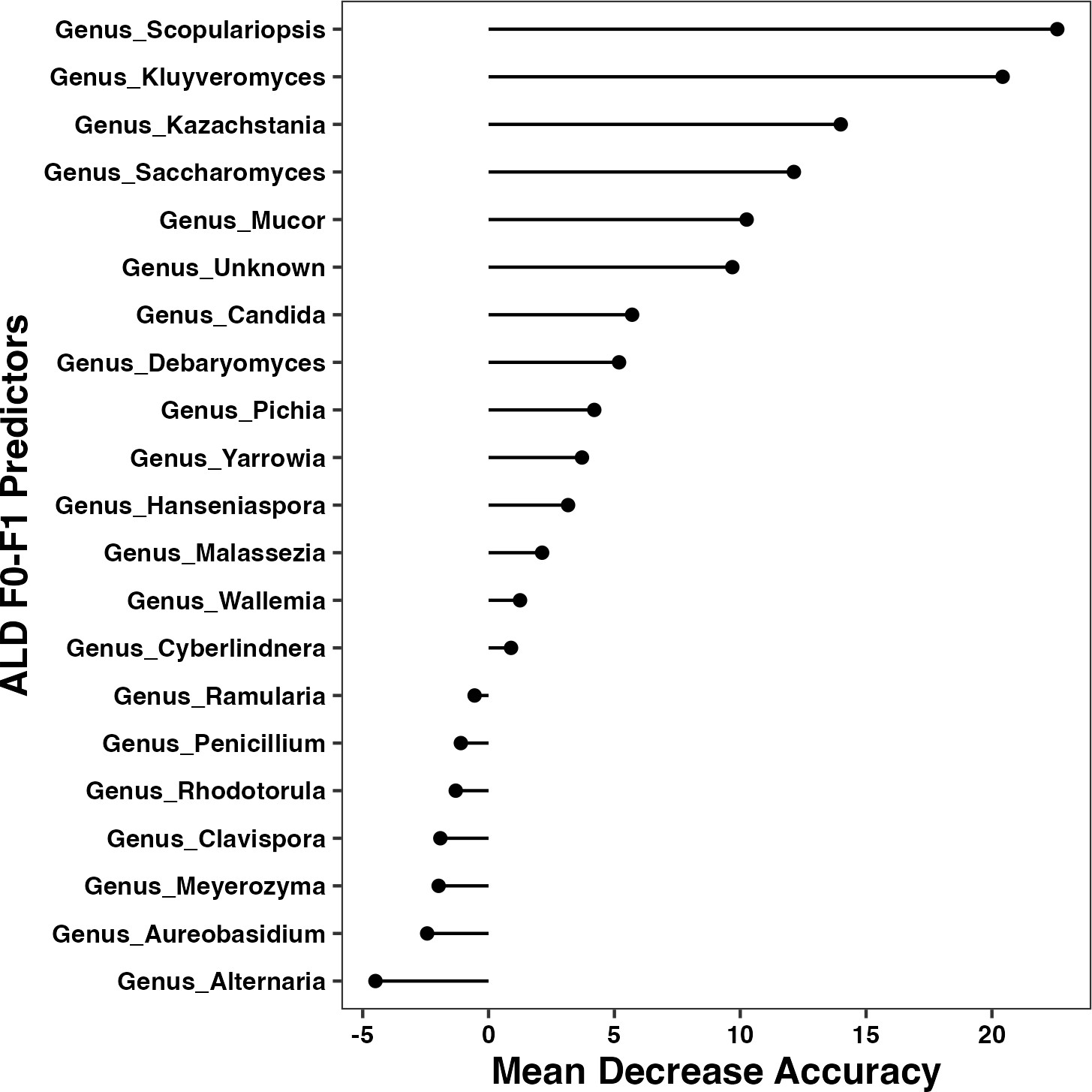

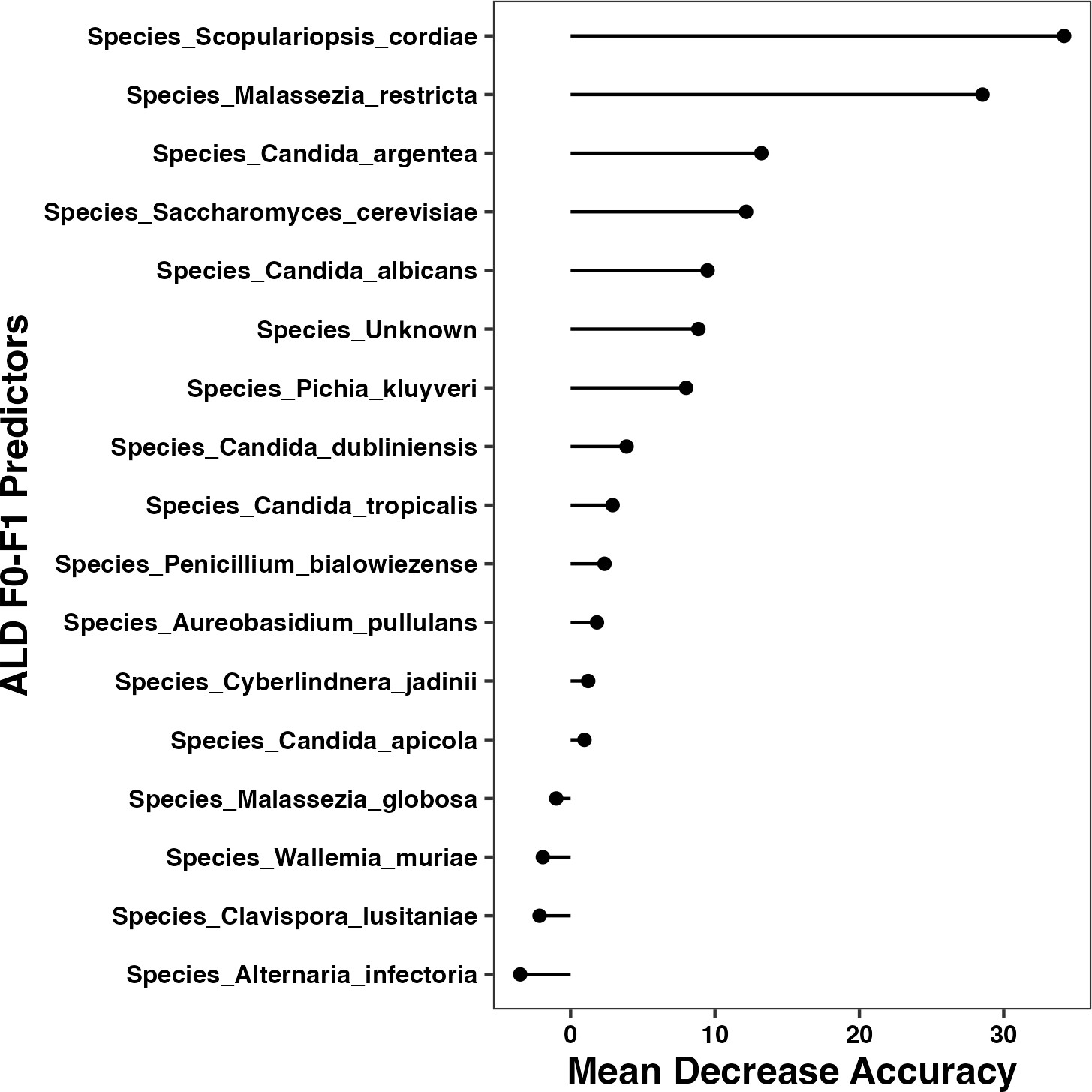


C D


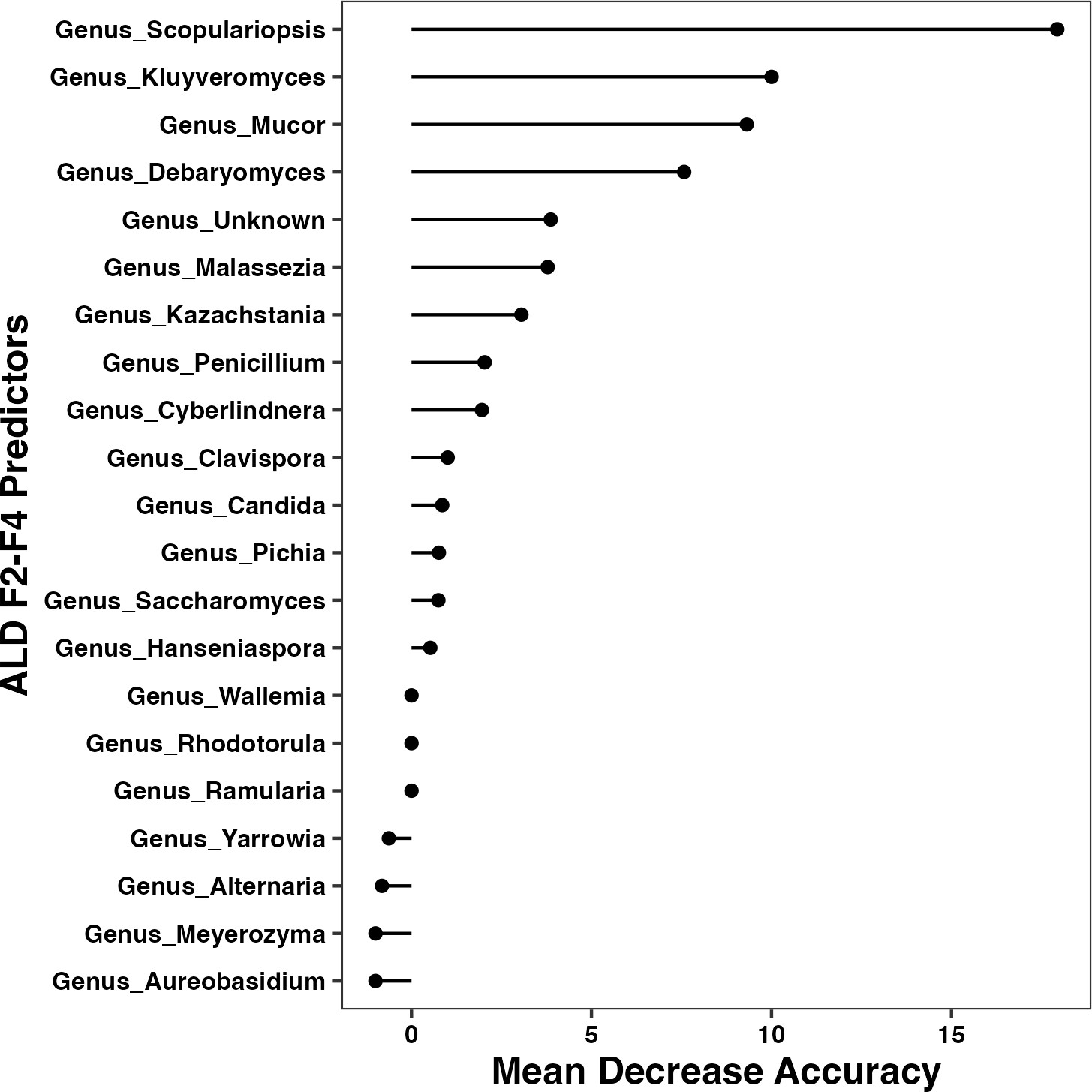

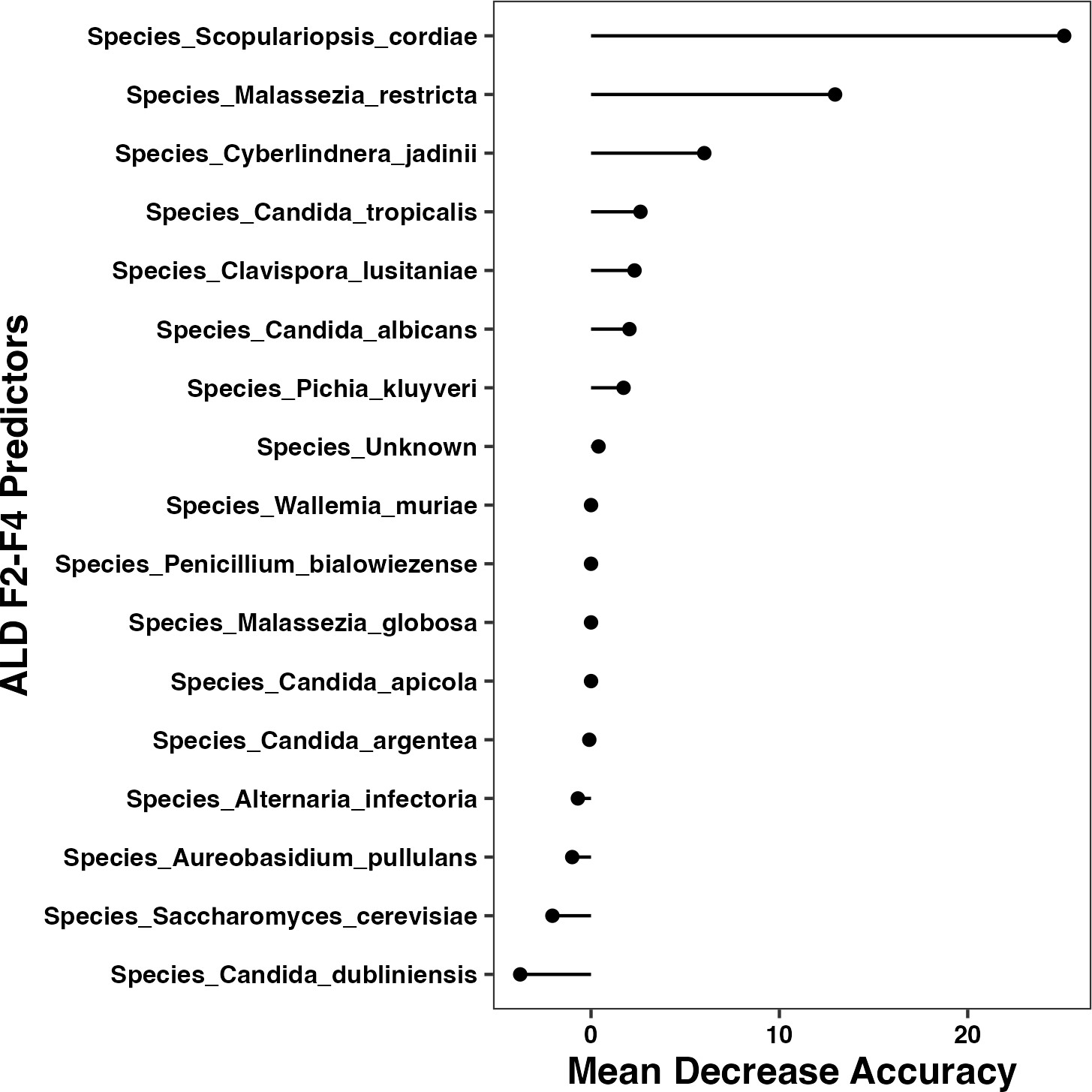

Supplement: Fungal signature differentiates Suppl Figures_R1.docx [file KGMI_A_2307586_SM5521.docx]
