# Supplementary material for: Fungal signature differentiates alcohol-associated liver disease from nonalcoholic fatty liver disease
Source: Gut Microbes. 2024 Feb 1;16(1):2307586. doi: 10.1080/19490976.2024.2307586 (PMC10841010; doi:10.1080/19490976.2024.2307586)
Supplement: Fungal signature differentiates ALD from NAFLD Suppl Material_R1.docx [file KGMI_A_2307586_SM5520.docx]

**Supplementary Figures**

**Supplementary Figure 1. Alpha diversity in ALD vs NAFLD vs healthy controls.** (A) Shannon index. (B) Inverse Simpson index. Healthy controls, n=34; ALD patients, n=58; NAFLD patients, n=78. ALD, alcohol-associated liver disease; HC, healthy controls; NAFLD, nonalcoholic fatty liver disease.

**Supplementary Figure 2. Most important fungal genera and species to identify ALD vs NAFLD stratified by fibrosis severity.** (A-B) Mean decrease accuracy by random forest analysis was determined for (A) fungal genera and (B) species to determine their respective feature importance for detecting ALD F0-F1 vs. NAFLD F0-F1. (C-D) Mean decrease accuracy by random forest analysis for (C) fungal genera and (D) species to determine their respective feature importance for detecting ALD F2-F4 vs. NAFLD F2-F4 (ALD F0-F1 patients, n=48; ALD F2-F4 patients, n=10; NAFLD F0-F1 patients, n=43; NAFLD F2-F4 patients, n=30). ALD, alcohol-associated liver disease; NAFLD, nonalcoholic fatty liver disease.
